# Benomyl modulates paracetamol bioaccumulation and endophytic microbiome diversity in zucchini

**DOI:** 10.1038/s41598-025-33977-6

**Published:** 2025-12-28

**Authors:** Huladduwa Mudiyanselage Chathurika Priyadarshani, Elżbieta Mierzejewska-Sinner, Bartosz Kózka, Joanna Giebułtowicz, Magdalena Urbaniak

**Affiliations:** 1 UNESCO Chair on Ecohydrology and Applied Ecology, Faculty of Biology and Environmental Protection, University of Lodz, Banacha 12/16, 90- 237 Lodz, Poland; 2 Department of Drug Chemistry, Pharmaceutical and Biomedical Analysis, Faculty of Pharmacy, Medical University of Warsaw, Banacha 1, 02-097 Warsaw, Poland

**Keywords:** Paracetamol, Benomyl, Plant physiology, Leaf endophytes, Phytoremediation, Environmental sciences, Environmental impact, Plant development, Plant ecology, Plant physiology, Plant symbiosis

## Abstract

Paracetamol, a widely used analgesic and antipyretic, has emerged as a prevalent environmental pollutant that poses risks to various organisms, including plants. Phytoremediation, particularly the use of Cucurbitaceae family plants, offers a promising, eco-friendly approach to mitigate such pollutants. This study investigated the bioaccumulation of paracetamol in zucchini (*Cucurbita pepo* cv. Atena Polka) and explored the role of the fungicide benomyl in modulating paracetamol bioaccumulation. Additionally, the impacts of these compounds on zucchini health (measured by biomass, chlorophyll content, and phenolic compound concentrations) and on the diversity of the endophytic microbiome (assessed via the Biolog EcoPlate™ and identification of isolated endophytes via 16S rRNA gene sequencing) were evaluated. Four treatments were assessed over a 28-day cultivation period: control (no pharmaceutical or fungicide), paracetamol (25 mg/kg) (P), paracetamol + benomyl (P + B), and benomyl alone (B). The results revealed that paracetamol accumulated significantly more in the roots of the P variant, but notably, the P + B variant presented a 24-fold increase in paracetamol concentration in aboveground tissues compared with the P variant. The bioaccumulation factors indicated that benomyl significantly enhanced the translocation of paracetamol to aerial parts in the P + B variant. Compared with the control, all the treatments reduced plant biomass, with the lowest values observed in the P variant. The chlorophyll content, in turn, was the highest in the P + B treatment, suggesting that benomyl helped mitigate oxidative stress. The levels of phenolic compounds, particularly flavonoids and phenylpropanoids, were elevated in the P variant, indicating a stress response. Additionally, the endophytic microbial community showed diminished diversity in the P variant. In the P + B variant, in turn, the microbial diversity measured via Biolog Ecoplates and 16S rRNA gene sequencing was greater. Our findings demonstrate that benomyl significantly enhanced the accumulation of paracetamol in the aerial parts of zucchini while promoting the resilience of plants and their associated endophytic bacteria and improving their potential for the phytoremoval of pharmaceutical pollutants.

**Supplementary Information:**

The online version contains supplementary material available at 10.1038/s41598-025-33977-6.

## Introduction

Pharmaceutically active compounds (PhACs) are emerging organic compounds that may significantly enhance our daily quality of life. The global consumption of PhACs continues to rise, driven by the spread of disease, changes in healthcare practices, and a growing global population^1^. Paracetamol (also known as acetaminophen) is a widely used analgesic and antipyretic PhAC that is recognized for its efficacy in symptom relief^2,3^. Paracetamol production reaches approximately 145,000 tons annually, and the drug is included in the World Health Organization’s essential medicines list; it is the most commonly used medication globally, with a consumption rate ranging from 3.6 to 47 g per person per year worldwide^4–6^. The COVID-19 pandemic has further increased medication consumption worldwide, with a notable 54% rise in searches for “paracetamol” compared with pre-pandemic levels^7^, making it the most over-the-counter sold pharmaceutical^8^.

The human body does not fully metabolize paracetamol, with 58–68% of the drug excreted unchanged into wastewater systems^9–11^. Studies from multiple countries, including Spain, France, the United Kingdom (UK), China, and the United States (US), have reported paracetamol concentrations in untreated wastewater ranging from 0.1 to 300 µg/L^12^. Conventional wastewater treatment processes are unable to eliminate paracetamol from inflow wastewater^13^. For example, research on four large-scale wastewater treatment plants in Japan reported an 80% removal efficiency^14^, whereas in European treatment facilities, the removal efficiency reached 85%^12^. Consequently, residual paracetamol persists in treated effluent, with concentrations reported in various countries: 0.16 µg/L in South Korea, 0.129–0.555 µg/L in the UK, 1.06 µg/L in the US, and 11.3 µg/L in France^9^. These residual levels ultimately contribute to environmental contamination, affecting aquatic ecosystems. Recent global study has detected paracetamol in rivers across 104 countries^15^. The average concentrations varied by region, ranging from 63.1 ng/L in Oceania through 291 ng/L and 395 ng/L in North America and Europe, respectively, to significantly higher levels of 2,790 ng/L, 5,660 ng/L, and 7,580 ng/L in Asia, Africa, and South America, respectively^15^. Owing to its widespread presence in aquatic environments, paracetamol is now among the top 10 priority PhACs for monitoring by the European Union^16^. Similarly, sewage sludge—the end product of the wastewater purification process—has been found to contain paracetamol at concentrations exceeding 300 µg/kg^17^, making it a potential source of this drug in the soil environment when used as a soil additive^18,19^.

The widespread presence of paracetamol in wastewater, sewage sludge, and surface water raises concerns about environmental contamination and hazards, as this PhAC can enter the soil‒plant system through irrigation with wastewater or fertilization with sewage sludge^18,19^. Research suggests that paracetamol can enter the food chain through plant uptake, with Cucurbitaceae crops (such as cucumbers, melons, and pumpkins) recognized for their ability to accumulate various pharmaceutical residues^20–22^. Cucurbitaceae plants are also known to absorb other environmental contaminants, including heavy metals, persistent organic pollutants (POPs), and emerging organic pollutants (EOPs)^23–25^. Zucchini, in particular, has shown a notable ability to accumulate high levels of organic contaminants in its above-ground tissues highlighting the phytoremediation potential of Cucurbitaceae crops in polluted soils^24,26–28^.

The presence of PhACs in the environment has raised significant concerns among scientists, sparking discussions about their potential impacts on ecosystem health. Studies suggest that even trace concentrations of drugs in water and soil can disrupt biological processes in organisms. This ongoing issue highlights the need for further research into the long-term effects of PhACs and the development of effective mitigation strategies. To address these challenges, it is essential to implement measures to monitor and control pharmaceutical levels in the environment and apply suitable mitigation solutions In this context, Cucurbitaceae crops, such as zucchini, offer a promising phytoremediation approach due to their ability to take up and sequester pharmaceuticals and other organic pollutants from contaminated soils. However, to safely harness this environmental benefit, it is essential to manage and monitor PhAC bioaccumulation in cucurbits, balancing food safety with their promising role in pharmaceutical phytoremediation. Interestingly, certain fungicides, such as oryzemate, can control the accumulation of organic pollutants in cucurbits by upregulating MLP genes, which govern the uptake of cyclic organic compounds in this family of plants^29–31^. Since MLPs bind strongly with aromatic compounds^32^, this process may also apply to PhACs, including paracetamol^33^. However, further research is essential to explore the phytoremediation potential and crop safety implications of fungicide use in this context.

Therefore, the overall aim of this study was to assess the potential for controlling the bioaccumulation of a selected PhAC in a cucurbit species to increase its phytoremediation ability. Specifically, this study examined the effects of benomyl, the active ingredient of the fungicide benomyl (also known as benlate), on the bioaccumulation of paracetamol in the roots and aboveground tissues of zucchini (*Cucurbita pepo* cv. Atena Polka). Given that the negative effects of paracetamol on plants have been demonstrated in numerous studies^34–36^, the effects of this drug and/or fungicide on zucchini health were also evaluated by measuring plant biomass, chlorophyll *a* and *b* contents, and phenolic compound concentrations. Finally, to assess the impact of the applied treatments on the quality of the plant’s endophytic microbiome—an integral component influencing the plant’s proper development and protection against environmental stress—the effects of both compounds on the functional and structural diversity of leaf-associated endophytic bacteria were examined. Our study is the first to examine the effects of the fungicide on paracetamol accumulation in zucchini, as well as the combined impact of both substances on the plant endophyte microbiome, highlighting the novelty of this research.

## Materials and methods

To ensure high replicability and avoid the influence of other pollutants or substances naturally present in the soil, this study utilized OECD artificial soil as the substrate medium. This soil, which is composed of sand, kaolin, and peat, is free of contaminants and is commonly employed as a reference in various soil toxicity tests, making it ideal for generating reliable and comparable experimental results^37,38^.

Zucchini (*Cucurbita pepo*, Atena Polka F1) was chosen for the experiment because of its potential to take up various pollutants on the basis of our previous studies^23,25,27,39^. The seeds were purchased from a certified supplier (W. Legutko).

### Experimental setup

Four treatment variants were tested in the experiment: (1) a control variant (C) with OECD soil planted with zucchini; (2) a paracetamol variant (P) with OECD soil spiked with paracetamol and planted with zucchini; (3) a paracetamol + benomyl variant (P + B) with OECD soil spiked with paracetamol, planted with zucchini, and sprayed with benomyl as described below; and (4) a benomyl variant (B) with OECD soil planted with zucchini and sprayed with benomyl as described below.

Four replicates (pots) were prepared for each variant. Two hundred cm^3^ pots were filled with 150 cm^3^ of OECD soil, and three zucchini seeds were introduced per pot. After one week, only one seedling was left in each pot, and the remaining plants were watered with paracetamol solution to achieve a concentration of 25 mg/kg. The applied paracetamol dose is consistent with concentrations used in previous studies assessing its effects on plants and microorganisms. For example, Badar et al.^40^ exposed spinach (*Spinacia oleracea* L.) to paracetamol concentrations ranging from 0 to 200 mg/L, while Fatima et al.^41^ investigated doses of 25–150 mg/L on the cyanobacterium *Nostoc muscorum*. Benomyl (DRE-C10490000; 250 mg/L) was sprayed on the aboveground parts of the plants according to the timeframe shown in Table S.1. All the samples were watered daily until they reached the water-holding capacity of OECD soil^23^. The experiment was conducted for 28 days in a growth chamber at 23 °C ± 0.5 °C and 60% w/v soil humidity^25,42,43^.

To maintain optimal growth conditions for zucchini, all the variants were fertilized with 60 mL of Florovit NPK 5-6-8 (10 mL of liquid fertilizer per 1.2 L of distilled water) on the 5th, 12th, 19th, and 26th days of cultivation.

### Determination of the paracetamol concentration in plant tissues

After lyophilization, 50 mg of aboveground plant tissue or 25 mg of root tissue was weighed in Lysing Matrix E tubes. Then, they were homogenized in a high-speed benchtop homogenizer (FastPrep-24 5Ga™, MP Biomedicals, 2 × 20s, 4 m/s, 4 °C) in a mixture containing 250 µL of an aqueous solution of internal standards and 600 µL of acetonitrile with formic acid (*v/v*, 999:1). Subsequently, 300 mg of ammonium acetate was added, and the samples were subjected to ultrasonication for 5 min, followed by orbital shaking for 15 min (1400 RPM). Finally, the mixtures were centrifuged at 9300×g for 10 min. The organic layer was diluted 6-fold with water. The final concentration of the internal standard was 20 ng/mL (acetaminophen-D4).

The prepared samples were analysed via high-performance liquid chromatography (HPLC) coupled with a mass spectrometer, i.e., HPLC-MS. HPLC analysis was performed via an Agilent 1260 Infinity instrument (Agilent Technologies, Santa Clara, CA, USA) equipped with a degasser, thermostatic autosampler, and binary pump and connected in series to an AB Sciex 4000 QTRAP mass spectrometer equipped with a Turbo Ion Spray source that was operated in both positive mode and negative mode (QTRAP^®^4000, AB SCIEX, Framingham, MA, USA). The curtain gas, ion source gas 1, ion source gas 2 and collision gas (all high purity nitrogen) pressures were set at 35 psi, 60 psi, 40 psi and “medium” instrument units, respectively; the ion spray voltage was set at 4500 V, and the source temperature was 600 °C.

Chromatographic separation was performed via a Hybrid Triple Quadrupole/Linear Ion trap mass spectrometer (QTRAP^®^4000, AB SCIEX, Framingham, MA, USA) with a Kinetex RP-18 column (100 mm × 4.6 mm, 2.6 μm) supplied by Phenomenex (Torrance, CA, USA). The column was maintained at 40 °C, and the flow rate was 0.5 mL/min. The mobile phase consisted of HPLC grade water with 0.2% formic acid as eluent A and acetonitrile with 0.2% formic acid as eluent B. The following gradient (%B) was used: 0 min. 10%, 1 min. 10%, 5 min. 90%, and 11 min. 90%. The injection volume was 10 µL.

The target compounds were analysed in multiple reaction monitoring (MRM) mode in positive ionization mode (ESI +), and two transitions between the precursor and most abundant fragment ions for each compound were monitored. The MRM-optimized conditions for quantitative transitions were as follows: acetaminophen *m/z* 152 > 110 (DP = 66 V, CE = 23 eV, CXP = 6 V); acetaminophen-D4: *m/z* 156 > 114 (DP = 66 V, CE = 25 eV; CXP = 8 V).

#### Phytoremediation potential of zucchini

The bioaccumulation factor (BAF) was calculated for the P and P + B variants via the following formula^44,45^:

$${\rm BAF}\,=\,{\rm C}_{\rm B}/{\rm C}_{\rm G}$$

where C_B_ represents the paracetamol concentration in the tissues of the plant roots/underground parts (mg/kg) and C_G_ represents the initial concentration of paracetamol in the soil (mg/kg).

### Plant physiological and biochemical analysis

#### Determination of biomass

After the plants were separated from the soil, the fresh biomasses of the roots, stems and leaves were weighed according to the methods of Urbaniak et al. and Rahman et al.^25,46^.

#### Determination of chlorophyll contents

Chlorophyll *a*, *b* and total chlorophyll contents were measured via 80% acetone extraction. Fresh leaves (0.2 g) were ground in a mortar. Two milliliters of 80% acetone was added, and the mixture was homogenized again. The suspension was transferred to a 25-mL graduated cylinder, and the total volume of the solution was increased to 10 mL with 80% acetone. The extraction mixture was mixed and left for 10 min to allow sedimentation of the homogenized plant material. Five milliliters of the supernatant was filtered through a soft filter into a test tube, and the absorbances of the samples were measured at 663 and 645 nm via a Thermo Scientific™ Multiskan™ Skyhigh Microplate Spectrophotometer^25,47,48^.

#### Determination of phenolic compound contents

Phenolic compounds (total phenols, phenylpropanoids, flavonols and anthocyanins) were determined in the leaves via 80% methanol extraction. Fresh leaves (0.15 g) were ground in a mortar. One milliliter of 80% methanol was added, and the mixture was homogenized again. The suspension was transferred to a 2 mL microcentrifuge tube and centrifuged at 5000 rpm for 15 min. The supernatant was transferred to a Falcon tube and brought to a volume of 1 mL with 80% methanol. A total of 0.25 mL of plant extract was mixed with 10 µL of 0.1% HCL and 2 mL of 2% HCL. The absorbances of the samples were measured at 280, 320, 360, and 520 nm via a Thermo Scientific™ Multiskan™ Skyhigh Microplate Spectrophotometer. The results are expressed as mg of standard per 100 g of fresh plant weight^49,50^.

### Analysis of leaf endophyte functional diversity via Biolog EcoPlates^TM^

#### Leaf surface sterilization and extraction

Surface sterilization and extract preparation were performed under a laminar flow under sterile conditions. A total of 3.5 g of fresh leaf sample was sterilized with 200 mL of 1 × 70% ethanol (1 min), 200 mL of 1 × 2.5% NaOCl + 0.1% Tween 20 (1 min), 200 mL of 1 × 70% ethanol (1 min), and 200 mL of 3x sterile distilled water (30 s). The sterility of the last wash was tested by inoculating 100 µl of the third rinse water on a Petri dish with undiluted LB medium^51,52^. The sterilized fresh leaf samples were homogenized via a sterile mortar and pestle, and 1% NaCl (up to 8 mL) was added. The extraction mixture was filtered through 100 μm mesh cell filters into a 50 mL Falcon tube. The volume was increased to 35 mL with 1% NaCl to prepare a 10^− 1^ dilution. A serial dilution was further prepared using 1% NaCl to obtain 10^− 2^ and 10^− 3^ dilutions of the leaf extracts^42,53^.

#### Endophyte functional diversity analysis

Endophyte functional diversity was analyzed via the Biolog EcoPlate^TM^ method. The analysis was performed under laminar flow under sterile conditions. In brief, 120 µL of 10^− 2^ diluted leaf extract was poured into each well of the microarray plates via a multichannel pipette. The plates were incubated at 27 °C for ten days. The optical density was measured at 590 nm at 24-hour intervals starting from time “0” via a Thermo Scientific™ Multiskan™ Skyhigh Microplate Spectrophotometer. The functional diversity of the endophytes was calculated on the basis of the consumption of 31 different carbon sources from 6 carbon groups (A-amines, AA-amino acids, CA-carboxylic acids, CCS-complex carbon sources, CH-carbohydrates, and PC-phosphate carbon) via Microsoft Excel 365^42,53^.

### Isolation of leaf endophytes and their identification via 16S rRNA gene sequencing

#### Leaf endophyte isolation

Endophytic bacteria from leaves were isolated under a laminar flow under sterile conditions. One hundred microliters of 10^− 1^, 10^− 2^, or 10^− 3^ dilutions (see point 2.4.1) of each variant were spread on LB media plates and incubated for 7 days at 27 °C. After incubation, the number of colonies on each plate was counted, and the number of colony-forming units (CFUs/mL) was calculated on the basis of the number of colonies counted in the 10^− 2^ dilution^54^. Distinct colonies from each plate were inoculated into LB medium plates with 50 µL of sterile MgSO_4_ via a sterile loop. The plates were stored in an incubator at 27 °C for 2–5 days. Pure colonies were inoculated into 2 mL sterile Eppendorf tubes containing 1 mL 869 liquid medium. Eppendorf tubes were incubated on a shaker at 27 °C for 2–10 days. The samples were subsequently centrifuged for 5 min at 8000 rpm and stored at 8 °C^55^.

#### DNA extraction and identification of bacterial isolates

Endophytic bacterial DNA was isolated via the GeneMATRIX Bacterial & Yeast Genomic DNA Purification Kit, Cat. No. E3580 (EURx Ltd. 80–297, Gdansk, Poland). The quality of the DNA samples was checked via a Nanodrop 2000 (Thermo Fisher Scientific), and all the concentrations were above 10 ng/µL. The extracted DNA samples were stored at -20 °C. The extracted genomic DNA was subjected to PCR using a 2720 Thermal Cycler. Universal primers for prokaryotes were used (27 F: 5′-AGAGTTTGATCCTGGCTCAG-3′, 1492R: 5′-GGTTACCTTGTTACGACTT-3′) for the analysis. PCR was performed for 25 cycles following a temperature gradient (initial denaturation: 98 °C, 180 s; denaturation: 98 °C, 10 s; annealing: 56 °C, 30 s; elongation: 72 °C, 60 s; and final elongation: 72 °C, 420 s)^56^.

Electrophoresis of the PCR products was conducted for 60 min at 100 V in 1x TBE buffer solution, and the products were visualized under UV light. The amplified products were sequenced via the same primers used for PCR amplification. Taxonomic classification was performed on the basis of the nucleotide alignments via BioEdit software and the BLAST tool at NCBI Web BLAST^56,57^.

### Data analysis

The data were analysed via Microsoft Excel 365 and PAST 4.0 software. On the basis of the normality of distribution, further statistical analyses were performed. The paracetamol accumulation, BAF, plant biomass, chlorophyll content, and phenolic compounds were compared via one-way analysis of variance (ANOVA) followed by Tukey’s HSD (honestly significant difference) post hoc test. Biolog EcoPlate^TM^ results, including Average Well Color Development (AWCD) for six carbon groups, and microbial catabolic diversity indices, were analyzed via the Kruskal‒Wallis test followed by Dunn’s post hoc test. All the statistical analyses were conducted at a significance level of *p* ≤ 0.05.

## Results

### Paracetamol concentration in zucchini

**Fig. 1:**
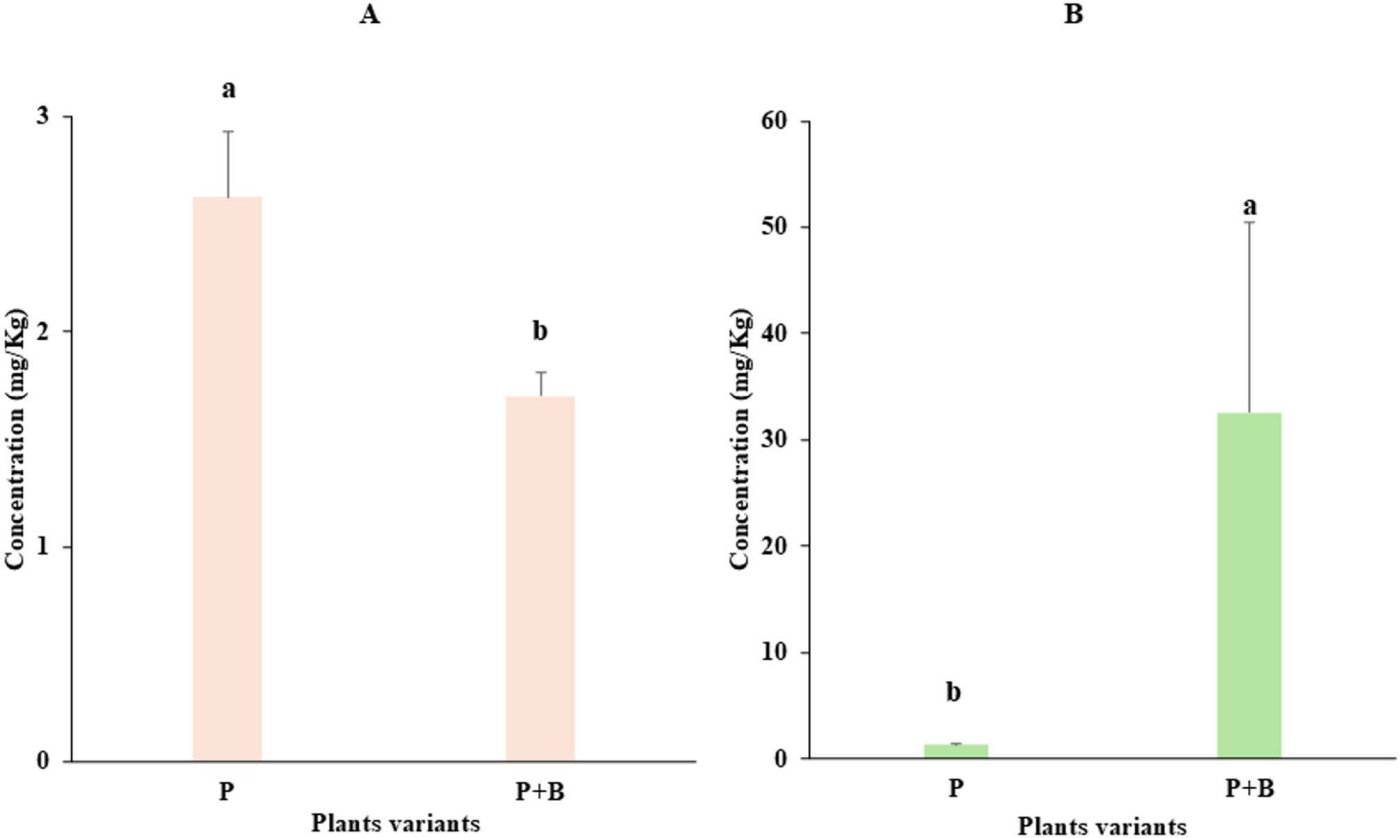
Mean paracetamol concentration. (**A**) Roots, *F*_(1,4)_ = 24.3, *p* = 0.007 and (**B**) aboveground parts, *F*_(1,4)_ = 9.12, *p* = 0.039. Lowercase letters indicate significant differences at *p* ≤ 0.05 according to Tukey post hoc test. The error bars indicate the standard deviation (*n* = 3).

The paracetamol concentrations in varieties with benomyl (P + B) and without benomyl (P) are shown in Fig. 1. In the roots, significantly greater concentrations were detected in the P variant (2.63 ± 0.31 mg/kg) than in the P + B variant (1.70 ± 0.11 mg/kg) (Fig. 1A). In contrast, in the aboveground parts of the plants—directly exposed to benomyl through spraying—the P + B treatment resulted in a significantly greater concentration (32.58 ± 17.91 mg/kg) than did the P treatment (1.35 ± 0.01 mg/kg), a 24-fold difference (Fig. 1B).

#### Bioaccumulation of paracetamol

Table 1 presents the BAF values for each plant variant, revealing significant differences among them. The P variant presented a greater BAF in roots (0.11 ± 0.01), indicating greater paracetamol retention below ground. In contrast, the P + B variant presented a notably greater BAF in aboveground tissues, exceeding 1.0 (1.30 ± 0.72), suggesting enhanced translocation and accumulation of paracetamol to the aerial parts. This elevated BAF in the P + B variant highlights a potential mechanism by which benomyl application influences contaminant distribution within plant tissues.

**Table 1:** Bioaccumulation factors (BAFs) for the root, *F*_(1,4)_ = 24.41, *p* = 0.007 and aboveground plant parts, *F*_(1,4)_ = 9.12, *p* = 0.039. Lowercase letters indicate significant differences at *p* ≤ 0.05 according to Tukey post hoc test.

Variants	BAF	
	Roots	Aboveground parts
P	0.11 ± 0.01^a^	0.05 ± 0.01^b^
P + B	0.07 ± 0.00^b^	1.30 ± 0.72^a^

### Plant physiology and biochemistry

#### Fresh biomass

**Fig. 2:**
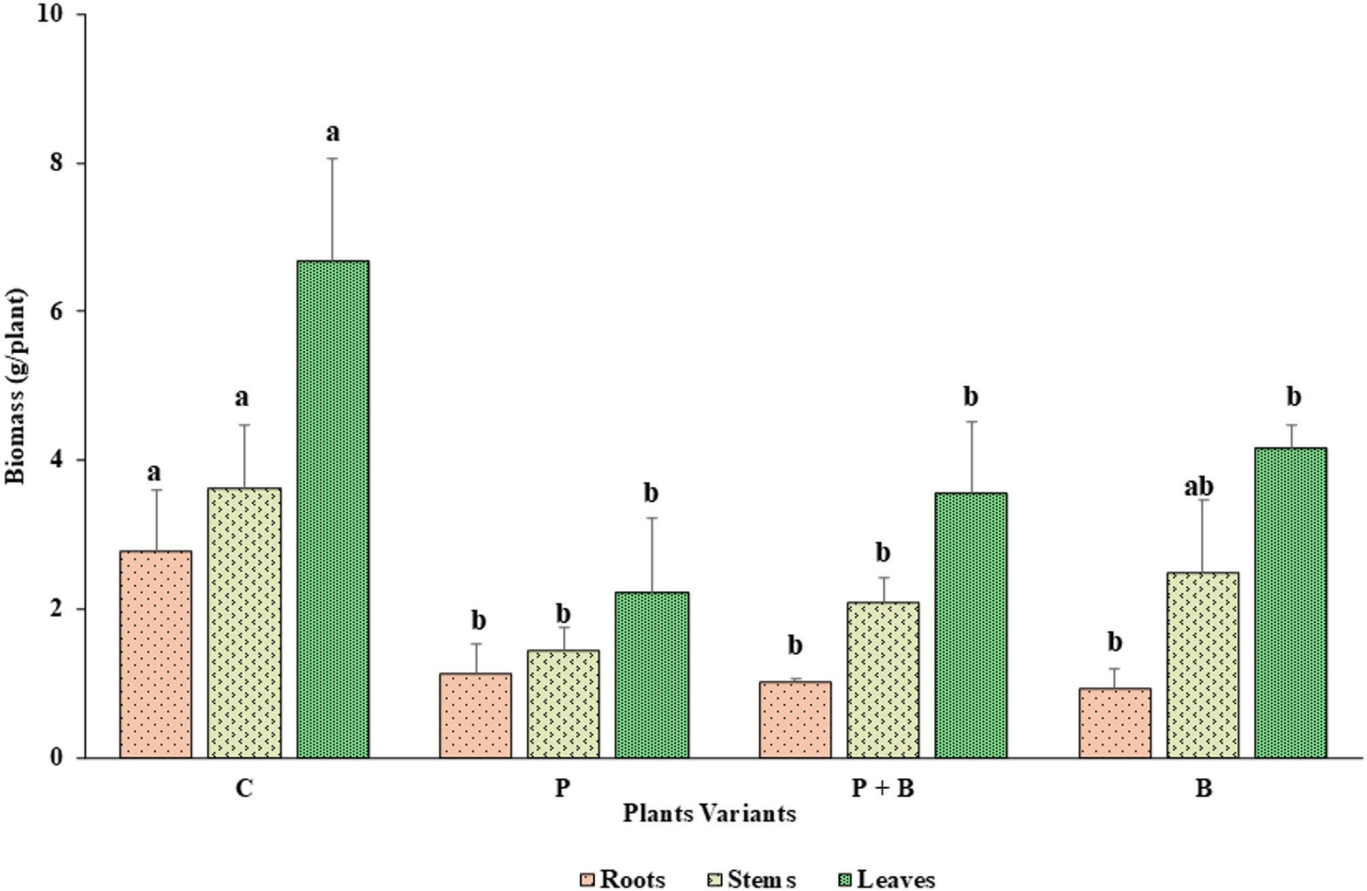
Average fresh biomass: roots, *F*_(3, 12)_ = 13.56, *p* = 0.0004; stems, *F*_(3, 12)_ = 7.103, *p* = 0.005; and leaves, *F*_(3, 12)_ = 14.24, *p* = 0.0003 for the plant variants control (C), paracetamol (P), paracetamol with benomyl (P + B) and benomyl (B). The error bars indicate the standard deviation (*n* = 4). Lowercase letters indicate significant differences between the same plant organs (i.e., roots, stems or leaves) at *p* ≤ 0.05 on the basis of the Tukey post hoc test.

Figure 2 presents the average fresh biomass of zucchini plants. The control group presented the highest biomass across all the plant parts: roots (2.78 ± 0.83 g/plant), stems (3.62 ± 0.85 g/plant), and leaves (6.69 ± 1.38 g/plant). In contrast, all the treatment groups presented a statistically significant reduction in biomass compared with that of the control group. The P variant presented the lowest biomass in stems (1.45 ± 0.30 g/plant) and leaves (2.23 ± 1.00 g/plant), with reductions of 60% and 67%, respectively, relative to the control (Fig. 2, Table S.2 in the supplementary materials). The root biomass in the P variant (1.13 ± 0.41 g/plant) was also significantly lower than that in the control, with the B variant resulting in the lowest overall root biomass (0.94 ± 0.20 g/plant). No statistically significant differences in root, stem, or leaf biomass were detected among the P, P + B, and B treatments. However, there was a noticeable trend of increasing biomass in stems and leaves in the order of P < P + B < B.

#### Chlorophyll contents

**Fig. 3:**
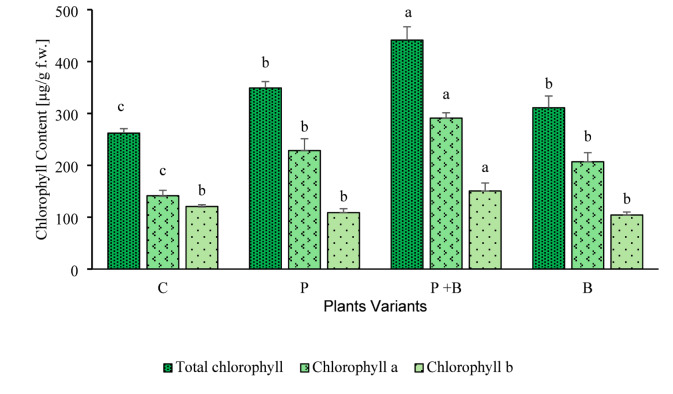
Average chlorophyll contents in zucchini leaves: total chlorophyll content, *F*_(3, 8)_ = 49.45, *p* = 0.001; chlorophyll *a* content, *F*_(3, 8)_ = 43.62, *p* = 0.002; and chlorophyll *b* content, *F*_(3, 8)_ = 15.27, *p* = 0.001 for plant variants. The error bars indicate the standard deviation (*n* = 3). Lowercase letters indicate significant differences at *p* ≤ 0.05 according to the Tukey post hoc test.

Figure 3 shows the average chlorophyll content in the zucchini leaves. The P + B treatment resulted in the highest chlorophyll content, with the total chlorophyll content reaching 441.44 ± 25.80 µg/g fresh weight (f.w.), the chlorophyll *a* content reaching 290.93 ± 10.44 µg/g f.w., and the chlorophyll *b* content reaching 150.51 ± 15.44 µg/g f.w. In contrast, the control group presented the lowest total chlorophyll (262.06 ± 8.60 µg/g f.w.) and chlorophyll *a* (141.39 ± 10.49 µg/g f.w.) contents. Interestingly, the P variant resulted in significantly greater total chlorophyll (349.17 ± 12.20 µg/g f.w.) and chlorophyll *a* (228.45 ± 22.85 µg/g f.w.) contents than did the control, whereas the chlorophyll *b* content remained statistically similar across the C, P, and B variants. Notably, the P + B variant demonstrated a substantial increase in chlorophylls compared with the P variant. Additionally, higher chlorophyll *a*/*b* ratios, averaging approximately 2, were observed in the P, P + B, and B variants (Tab. S.3 in the supplementary materials). The control treatment, however, had the lowest chlorophyll *a*/*b* ratio (1.17 ± 0.11), indicating a distinct chlorophyll composition relative to that of the treated plants.

#### Phenolic compound contents

**Fig. 4:**
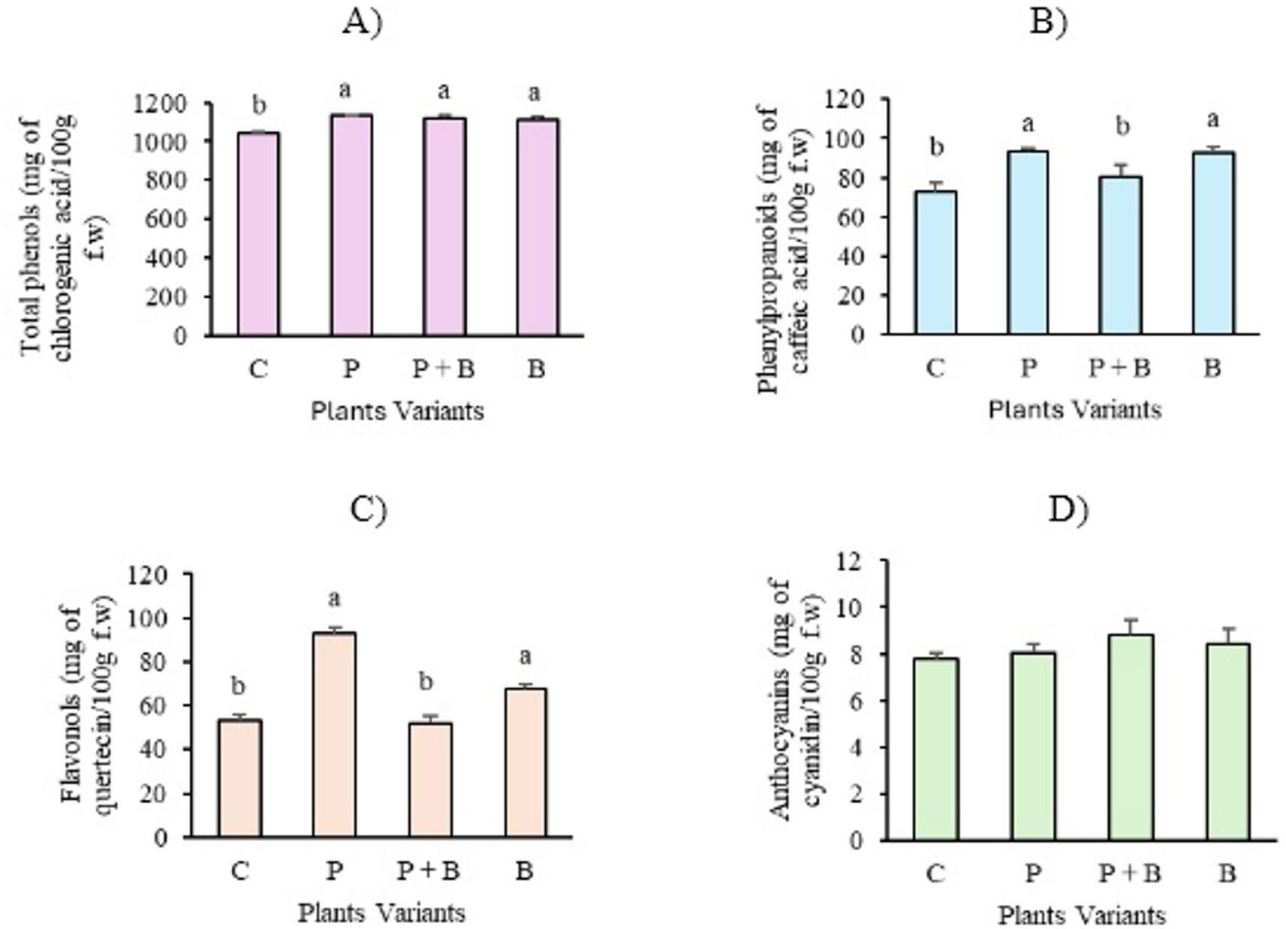
Average phenolic compounds in zucchini leaves: (**A**) total phenols *F*_(3, 8)_ = 30.94, *p* = 0.009; (**B**) phenylpropanoids *F*_(3, 8)_ = 17.49, *p* = 0.0007; (**C**) flavonols *F*_(3, 8)_ = 23.9, *p* = 0.0002; and (**D**) anthocyanins *F*_(3, 8)_ = 2.163, *p* = 0.17. The error bars indicate the standard deviation (*n* = 3). Lowercase letters indicate significant differences at *p* ≤ 0.05 according to the Tukey post hoc test.

Figure 4 displays the average phenolic compound levels in the zucchini leaves. The highest total phenolic concentrations were observed in the P variant (1131.32 ± 5.65 mg/100 g f.w.), closely followed by the P + B variant (1117.04 ± 19.17 mg/100 g f.w.) and the B variant (1114.93 ± 12.77 mg/100 g f.w.). In contrast, the control group presented the lowest total phenol content, at 1042.11 ± 7.94 mg/100 g f.w. (Fig. 4A). Notably, the P and B variants presented significantly higher and statistically similar levels of both phenylpropanoids and flavonols, whereas the control and P + B variants presented the lowest concentrations of these compounds (Fig. 4B and C). No significant differences in anthocyanin levels were observed across the variants.

### Diversity of plant endophytes via Biolog EcoPlate^TM^ analysis

**Fig. 5:**
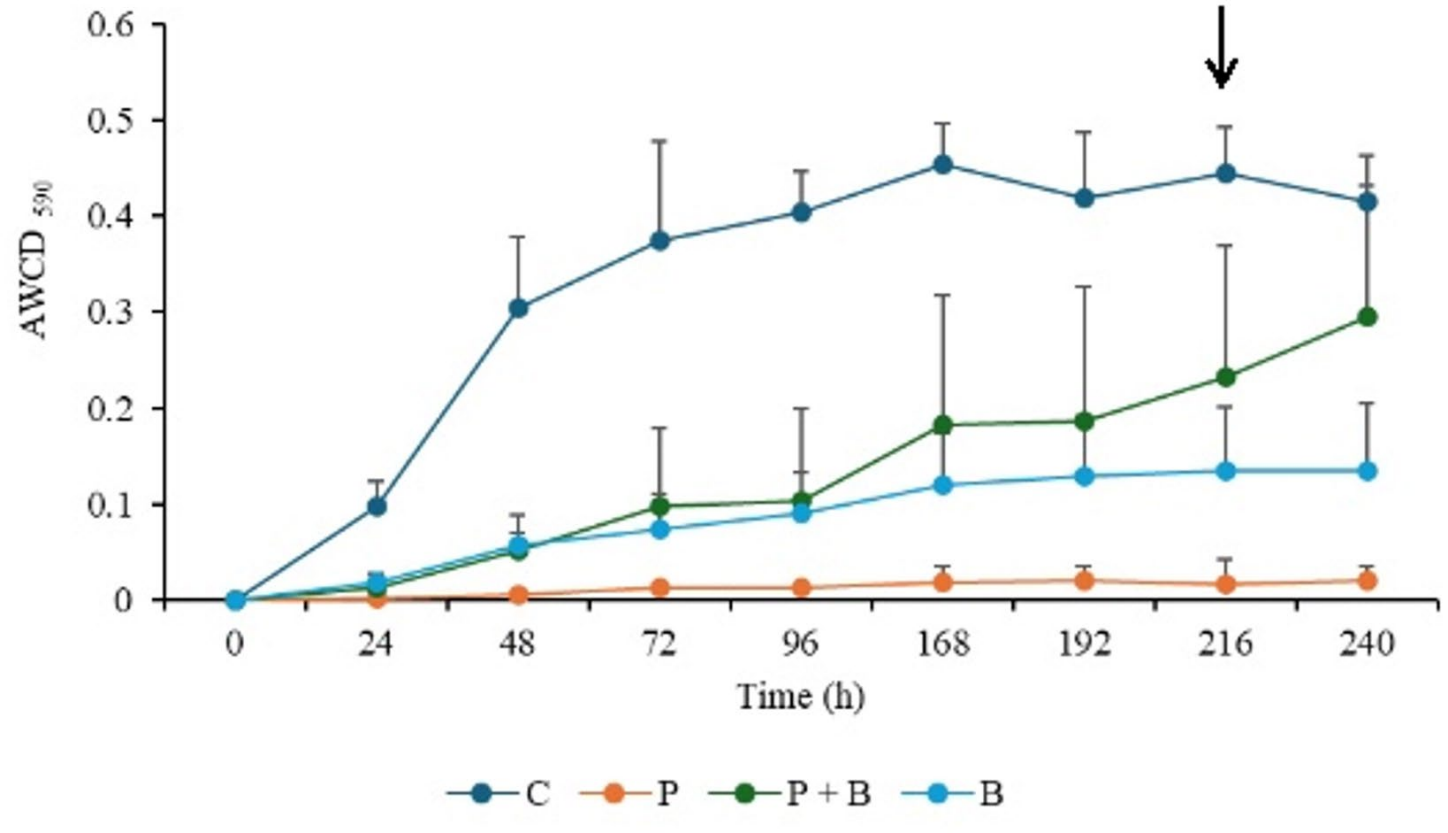
Dynamics of the AWCD index over a 240-hour incubation period. The black arrow indicates optimum carbon substrate utilization for all the variants after 216 h of incubation.

The functional diversity of the leaf endophyte microbial community was assessed via AWCD. Figure 5 shows the changes in AWCD over a 240-hour incubation period. The control group presented the highest AWCD values, peaking at 168 h. In contrast, the P variant presented the lowest AWCD, whereas the P + B variant presented a significant increase compared with the P variant. Notably, the overall optimal carbon substrate utilization was observed at 216 h across all the variants, which was selected for further calculations.

**Fig. 6:**
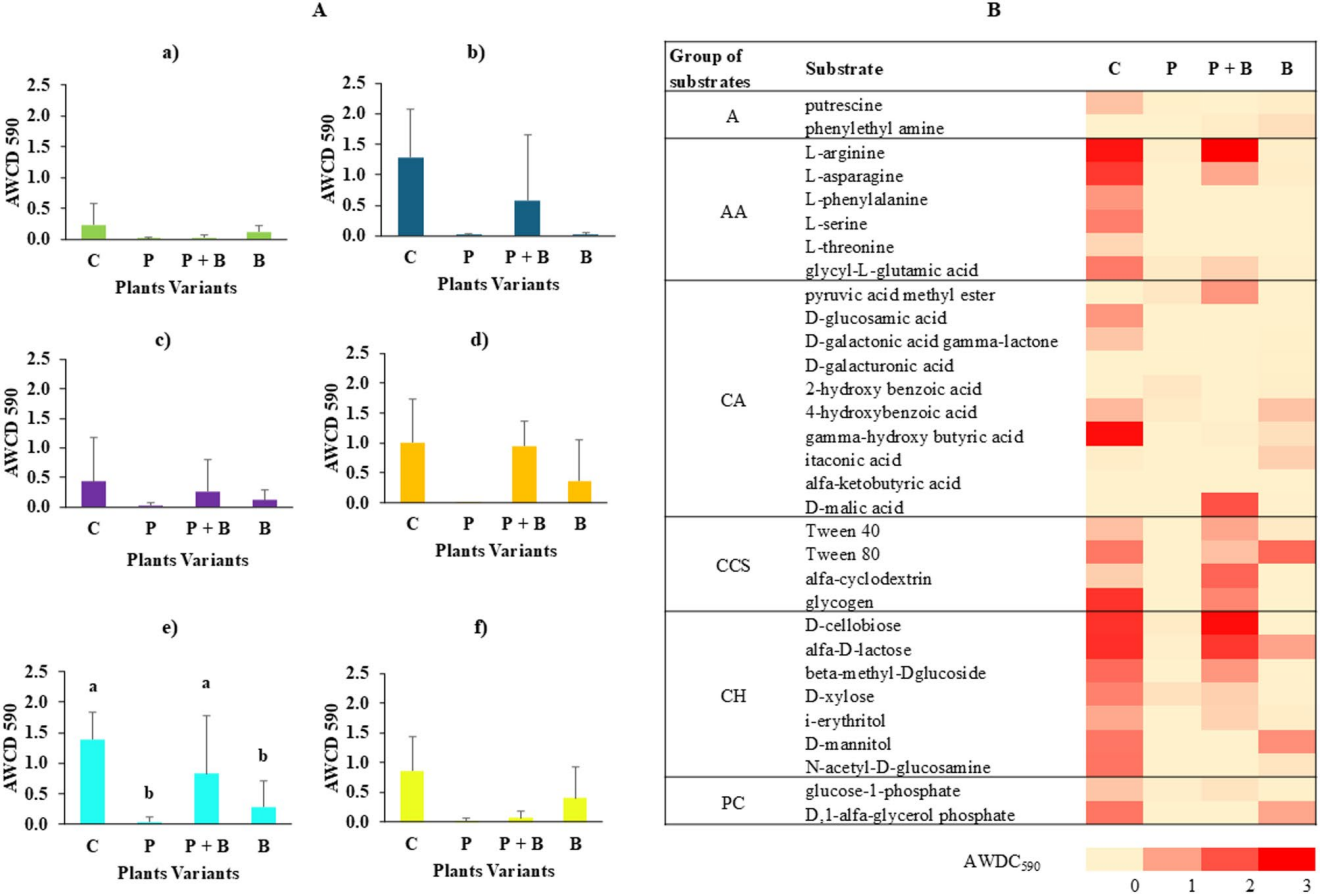
(**A**) AWCD for the six groups for paracetamol treatment after 216 h in the EcoPlate™ assay: (**a**) amines (A), (**b**) amino acids (AAs), (**c**) carboxylic acids (CAs), (**d**) complex carbon sources (CCSs), (**e**) carbohydrates (CHs), *p* = 0.02 and (**f**) phosphate carbon (PC). Lowercase letters indicate a significant difference at *p* ≤ 0.05 according to Dunn’s post hoc test. (**B**) Heatmap of 31 carbon substrates after 216 h of incubation.

Figure 6A presents the microbial consumption of the six main carbon groups after a 216-hour incubation period. Among the variants, only carbohydrate consumption exhibited statistically significant differences. Compared with the P + B variant (AWCD; 0.83 ± 0.94), the control group presented greater and statistically similar carbohydrate utilization (AWCD; 1.39 ± 0.45). In contrast, lower carbohydrate utilization rates were recorded for the B (AWCD; 0.29 ± 0.42) and P (AWCD; 0.04 ± 0.07) variants (Fig. 6A). While most carbon sources did not significantly differ, the control consistently demonstrated the highest utilization across all carbon groups, whereas the P variant presented the lowest utilization. Additionally, a trend toward increased carbon utilization in the P + B variant was observed compared with that in the P variant.

Figure 6B shows a heatmap of 31 carbon substrates following 216 h of incubation. In group A, only the control exhibited minimal usage of putrescine (AWCD = 0.48). In the AA group, both the control and the P + B variants efficiently consumed L-asparagine, with AWCD values of 2.25 and 2.45, respectively. The control also utilized L-asparagine, L-serine, and glycyl-L-glutamic acid, with AWCD values of 1.88, 1.18, and 1.22, respectively. Conversely, the P + B variant demonstrated minimal consumption of L-asparagine (AWCD = 0.75). In the CA group, the control displayed the highest substrate consumption for gamma-hydroxybutyric acid (AWCD = 2.32), whereas the P + B variant utilized D-malic acid with an AWCD of 1.63. A lower substrate consumption was noted in the control for D-glucosamine acid (AWCD = 0.93) and D-galactonic acid gamma-lactone (AWCD = 0.45) and in the P + B variant for pyruvic acid methyl ester (AWCD = 0.94).

In the CCS group, substrates were significantly consumed by both the C and P + B variants. For the CH group, D-cellobiose and alpha-D-lactose exhibited considerably greater utilization in both the C and P + B variants. In the PC group, the control consumed D,1-alpha-glycerol phosphate with an AWCD of 1.27. Overall, microbial metabolism among the substrates increased in the following order: P < B < P + B < C. The P variant demonstrated the lowest substrate usage, failing to consume 18 substrates, which indicates minimal metabolic activity. Interestingly, compared with the P variant, the P + B variant resulted in increased microbial metabolism.

#### Microbial catabolic diversity

Table 2 presents the microbial catabolic diversity indices derived from the Biolog EcoPlate™ data. The Shannon‒Weaver diversity (H′) index differed significantly among the variants, ranging from 0.17 to 2.30. The H′ index was highest in the control group (2.30 ± 0.08), whereas it was lowest in the P treatment group (0.17 ± 0.12). Compared with the P variant, the P + B variant had a substantially greater H′ index (1.24 ± 0.90). The Shannon evenness index (E) also varied significantly among the variants, ranging from 0.08 to 0.92. The control group presented the highest E value (0.92 ± 0.06), whereas the P variant had the lowest (0.08 ± 0.06). Interestingly, the E index also significantly increased in the P + B variant (0.64 ± 0.12) compared with that in the P. In contrast, the substrate richness index (S) did not significantly differ across all the variants, with no value for the P variant.

**Table 2:** Microbial catabolic diversity indices for the paracetamol treatment after 216 h of incubation: Shannon‒Weaver diversity index (H′); *p* = 0.03, Shannon evenness index (E); *p* = 0.02 and substrate richness index (S). Lowercase letters indicate significant differences at *p* ≤ 0.05 according to Dunn’s post hoc test.

Plants variants	H’ (± SD, *n* = 3)	E (± SD, *n* = 3)	S (± SD, *n* = 3)
C	2.30 ± 0.08 ^a^	0.92 ± 0.06 ^a^	10.67 ± 1.15^a^
P	0.17 ± 0.12 ^c^	0.08 ± 0.06 ^c^	0.00 ± 0.00^c^
P + B	1.24 ± 0.90 ^ab^	0.64 ± 0.12 ^ab^	6.33 ± 4.62^ab^
B	0.83 ± 0.31 ^bc^	0.32 ± 0.13 ^bc^	3.67 ± 1.53^bc^

### Diversity of isolated plant endophytes on the basis of 16S rRNA gene sequencing

In examining the microbial colony formation of the leaf endophytes, the highest CFUs for the 10^− 1^ dilution of leaf extracts were observed in the P + B variant, with a value of 25.5 CFU × 10²/mL. In contrast, the control group presented the lowest CFU at 9.5 × 10²/mL. Notably, no colony formation was detected in the P variant (Table S.4 in the supplementary materials).

PCR analysis confirmed the presence of the 16S rRNA gene (1465 base pairs) in DNA samples extracted from endophytic microbial colonies isolated from the C, P + B, and B variants. Owing to the absence of microbial colonies in the P variant, the 16S rRNA gene was not detected.

Further 16S rRNA sequencing analysis led to the identification of leaf endophytic bacteria, which are summarized in Table 3. The sequencing analysis revealed three distinct bacterial phyla: Bacillota, Actinomycetota, and Pseudomonadota. Bacillota was the most dominant, appearing in the C, P + B, and B variants. Pseudomonadota was found in the C and B variants, whereas Actinomycetota was present only in the P + B variant. *Bacillus* was the most prominent bacterial genus among the variants and was found in the C, P + B, and B variants. The control had the most bacterial genera (6 genera), followed by the P + B and B variants (5 genera per group).

**Table 3:** Identified endophytic bacteria up to the genus level, query length and percent identity for each variant.

Variants	Phylum	Class	Family	Genus	Query length	Percent identity (%)
C	Pseudomonadota Bacillota Bacillota Bacillota Bacillota Bacillota	Gammaproteobacteria Bacilli Bacilli Bacilli Bacilli Bacilli	Enterobacteriaceae Bacillaceae Bacillaceae Bacillaceae Bacillaceae Bacillaceae	*Enterobacter* *Bacillus* *Bacillus* *Gottfriedia* *Bacillus* *Bacillus*	803 817 801 402 524 407	100 100 100 99.5 100 90.2
P	ND	ND	ND	*ND*	ND	ND
P + B	Bacillota Bacillota Actinomycetota Bacillota Actinomycetota	Bacilli Bacilli Actinomycetia Bacilli Actinomycetia	Bacillaceae Bacillaceae Microbacteriaceae Bacillaceae Microbacteriaceae	*Bacillus* *Bacillus* *Microbacterium* *Bacillus* *Microbacterium*	748 764 488 490 534	100 100 99.6 97.4 99.4
B	Bacillota Bacillota Bacillota Pseudomonadota Bacillota	Bacilli Bacilli Bacilli Gammaproteobacteria Bacilli	Bacillaceae Bacillaceae Bacillaceae Erwiniaceae Bacillaceae	*Bacillus* *Bacillus* *Neobacillus* *Pantoea* *Bacillus*	753 813 767 500 572	100 100 100 99 99.8

ND: Not detected.

## Discussion

### Paracetamol accumulation in plant tissues

The results obtained in this study (see Fig. 1A and B) demonstrated the effects of paracetamol uptake and accumulation in zucchini. Similarly, paracetamol has been detected in zucchini and cucumber tissues^24,26^, as well as in various edible plants, including spinach, cabbage, celery^58^, jute mallow^59^, maize^35^, and lettuce^60^. According to the above studies, paracetamol uptake and accumulation are primarily concentration- and organ dependent. However, pollutant accumulation in plants can depend on various factors, one of which is the influence of other organic substances. In our study, paracetamol accumulation significantly decreased in the roots but increased in the leaves when the plants were treated with benomyl. This trend was further supported by the BAF, which was significantly greater in the aboveground parts when the plants were treated with both paracetamol and benomyl (see Table 1).

Our results align with findings from Chitose et al.^29^, who reported that the agrochemical oryzemate promoted the uptake of hydrophobic organic pollutants by zucchini grown in highly contaminated soil. The underlying mechanism may involve the translocation of organic pollutants into xylem vessels, which is critical for the transfer of pollutants to aerial parts of the Cucurbitaceae family^24,31,39,61^. Certain compounds, such as pesticides and fungicides, have been shown to increase this process by increasing the expression of *MLP* genes, which are crucial for the uptake and transport of organic pollutants in Cucurbitaceae^29^.

Similarly, in this study, benomyl may facilitate the formation of MLP-paracetamol complexes, enabling the transport of paracetamol from roots to aerial parts. This mechanism could explain the observed reduction in the paracetamol concentration in the roots (see Fig. 1A) and the corresponding increase in the aerial parts (see Fig. 1B). However, further research is needed to thoroughly elucidate the molecular mechanisms behind this phenomenon and its potential practical implications.

### Influence of paracetamol and/or fungicide on plant physiology and biochemistry

Plant biomass is a widely recognized indicator of environmental stress for assessing phytotoxicity^62^. Numerous studies have documented the effects of various organic pollutants on the growth and biomass of cucurbits, often through alterations in their biometric and physiological parameters^25,42^. Paracetamol has been shown to induce oxidative stress in plants, damage cellular components, reduce the leaf area, and inhibit water and nutrient absorption by shortening the root system. These effects collectively limit plant growth and biomass^34,63,64^.

In our experiment, the fresh biomass of zucchini—including roots, stems, and leaves—significantly decreased following exposure to 25 mg/kg paracetamol (Fig. 2, Table S.2). Comparable biomass reductions have been reported in cucumber exposed to 10 mg/L paracetamol^26^ and maize exposed to 310–1240 mg/L paracetamol^35^. Zezulka et al.^64^ reported dry weight reductions of 37% in pea and 23% in maize, both at 10 mg/L paracetamol. Conversely, lower paracetamol concentrations had no effect on the biomass of *Solanum nigrum* at 0.25 and 0.5 mg/L^65^ or on that of lettuce at 0.1–5 mg/L^60^. These findings align with those of Badar et al.^58^, who reported that biomass declines become more pronounced in plants exposed to paracetamol concentrations exceeding 50 mg/L. Compared with the control, benomyl treatment reduced plant biomass (see Fig. 2), which aligns with the findings of Kara et al.^66^, who reported that benomyl can negatively impact plant growth and biomass. Interestingly, the P + B treatment resulted in increased biomass compared with the paracetamol-only treatment (Fig. 2), suggesting that benomyl might mitigate the phytotoxic effects of paracetamol in zucchini, although the exact mechanisms remain unclear. In contrast, Fujita et al.^30^reported no significant differences in the fresh weight of the aerial parts of zucchini grown in soil contaminated with hydrophobic pollutants and treated with pesticides.

In addition to plant biomass, chlorophyll pigments also serve as functional indicators of plant health and productivity when evaluating the responses of cucurbits to contaminants^25,62,64^. In this study, the total chlorophyll content, chlorophyll *a* content, and chlorophyll *a*/*b* ratio increased with paracetamol treatment compared with those of the control (see Fig. 3 and Table S.3). These results align with the findings of Badar et al.^58^ and Kudrna et al.^67^, who reported increased chlorophyll levels in spinach at 100–200 mg/L paracetamol and in lettuce at 500 µM–5 mM paracetamol. Increased pigment levels may reflect adaptive or stress-response mechanisms in plants involving enzymatic, genetic, or metabolic adjustments^36,68,69^. However, despite increased chlorophyll levels, certain plants, such as spinach, presented reduced photosynthetic efficiency due to impaired photosystems I and II and disrupted electron transport under paracetamol exposure^58^. Paracetamol can also degrade chlorophyll enzymatically or inhibit pigment biosynthesis^60,70,71^, although some plants exhibit no adverse response even at high concentrations^72^. An increase in the chlorophyll *a* content might explain the observed increase in the chlorophyll *a*/*b* ratio. Benomyl treatment further increased the chlorophyll content and the chlorophyll *a*/*b* ratio in our study (see Fig. 3 and Table S.3). Some studies reported increased photosynthesis in benomyl-treated plants, whereas others reported reduced efficiency^73^. The highest chlorophyll levels and *a*/*b* ratios occurred in the P + B variant (see Fig. 3 and Table S.3), suggesting that the combination of paracetamol and benomyl may reduce phytotoxicity or induce metabolic changes that increase the chlorophyll content.

Another indicator of plant health and the effects of various stress factors are phenolic compounds. Phenolic compounds, secondary plant metabolites, play vital roles in physiological and biochemical functions, particularly in defence against biotic and abiotic stressors. These potent antioxidants effectively neutralize oxidative stress and often accumulate in response to environmental challenges^74,75^. In our study, total phenols, phenylpropanoids, and flavonols were significantly elevated in response to paracetamol treatment (Fig. 4). These findings indicate that paracetamol exposure disrupts normal biosynthetic pathways, leading to the accumulation of phenolic compounds in zucchini. These findings are consistent with those of Kummerová et al.^76^, who reported a substantial increase in polyphenol content in *Lemna minor* exposed to 100 µg/L paracetamol. On the other hand, higher concentrations, such as 0.5 mg/L, did not affect total phenols in lentil or broad bean, although some species presented reductions. Variations in flavonoid production are influenced by NSAID concentrations, plant defense mechanisms, and the duration of oxidative stress^71^. Phenolic compound levels were also significantly elevated in the B treatment (see Fig. 4), likely due to oxidative stress induced by fungicides^77^. Interestingly, the phenylpropanoid and flavonols concentrations were significantly lower in the P + B treatment group than in the paracetamol alone group and were statistically similar to those in the control group (see Fig. 4). These findings suggest a potential interaction between paracetamol and benomyl that may reduce oxidative stress.

### Influence of paracetamol and/or fungicides on the plant endophytic microbial community

Endophyte-assisted phytoremediation holds great promise for environmental decontamination, as many endophytes possess pollutant-degrading capabilities. However, research on endophytic microbes in plants grown in pharmaceutical-contaminated soil remains limited. This contrasts with the more extensively studied areas of general endophyte research, rhizosphere microorganisms, and the effects of pollutants on microbial communities in soil, water, or pure cultures. Most available studies emphasize the impact of contaminants on plant physiology and biochemistry^78,79^. Our study may be the first to examine the effects of paracetamol and/or fungicide on leaf endophytes in Cucurbitaceae. This unique focus makes direct comparisons with findings from other studies challenging, highlighting the novelty of our research.

The Biolog EcoPlate™ assay is a widely used tool for indirectly assessing microbial functional diversity by analyzing the utilization of 31 carbon substrates^80^. Despite its broad application, studies using the Biolog EcoPlate^TM^ to evaluate the metabolic profiles of plant-associated microbiomes remain limited^81^. The assay generates AWCD values, which indicate microbial metabolic activity; higher AWCD values correspond to greater activity^78^. In our study, AWCD analysis revealed the highest microbial metabolic activity in the control samples, whereas the paracetamol-treated samples presented significantly reduced activity. These findings suggest that the applied dose of 25 mg/kg paracetamol can negatively impact the metabolic activity of zucchini leaf endophytic microbes. Similarly, Cycoń et al.^82^ reported that PhACs can disrupt microbial metabolism by inhibiting catabolic reactions and damaging cellular integrity. Additionally, Kovacs et al.^78^ reported reduced microbial abundance in the rhizosphere of tomato plants exposed to PhACs such as diclofenac, ketoprofen, and ibuprofen. Although endophytes may be less vulnerable than rhizosphere microbes are, paracetamol accumulation within plant tissues could still alter the endophytic community composition and activity, affecting their functional roles in the host plant. Compared with the control, the benomyl-treated variant also presented lower microbial metabolic activity, possibly due to fungicide-induced effects on endophytic fungi, which are crucial for maintaining the microbial metabolic balance. Interestingly, the AWCD values were greater in the benomyl treatment group than in the paracetamol alone group, suggesting that paracetamol has a greater effect on the endophyte community.

Microbial communities often prefer carbohydrates and carboxylic acids because of their ease of digestion^83^. In our study, a significant reduction in carbohydrate consumption was observed in the paracetamol and benomyl treatment groups compared with the control group. While the overall carbon consumption differences were not statistically significant, the paracetamol treatment resulted in a noticeable decline. This reduction could be attributed to adverse effects on microbial communities, such as decreased abundance^78^ or cellular damage^82^. A healthier microbial community likely supports more efficient carbon substrate utilization. Heatmap analysis further revealed distinct carbon consumption patterns among the treatments. The control group utilized a broader range of carbon sources, reflecting more robust microbial activity. In contrast, P treatment significantly reduced carbon source utilization. These findings are consistent with those of Kovacs et al.^78^, who reported restricted carbon substrate utilization by tomato rhizosphere microbes under diclofenac exposure. Similarly, our results indicate that paracetamol exposure limits the diversity of carbon sources utilized by endophytes. This was additionally confirmed by microbial catabolic diversity indices^84^. Our results demonstrated a significant negative impact of paracetamol on the catabolic diversity of zucchini endophytes, which was partially mitigated by the combined application of paracetamol and benomyl (P + B) (see Table 2).

In summary, compared with the individual P and B treatments, the combined P + B treatment significantly increased the functional diversity of the zucchini endophytes. This was evidenced by higher AWCD values, increased carbon substrate utilization, and elevated microbial catabolic diversity indices.

The above results were further confirmed via culture-dependent identification of endophytes via 16S rRNA sequencing. In this case, no CFUs were observed in the P treatment (Tab. S.4), while the combined P + B treatment had the highest CFU count, indicating that their joint application might enhance the endophytic microbial community in zucchini and aligning with earlier discussions on how combined treatments can influence microbial functional diversity. The detrimental effect of paracetamol on plant endophytes aligns with studies reporting the presence of 16S rRNA genes in microbial isolates from plants and rhizosphere microbes exposed to PhACs, including paracetamol^58,85–88^.

Studies have shown that endophytes possess diverse metabolic genes, which play crucial roles in degrading pollutants, enhancing bioremediation, and promoting plant growth in contaminated soils^43,89–93^. Bacterial families such as Pseudomonaceae, Burkholderiaceae, Bacillaceae, and Enterobacteriaceae are particularly abundant in polluted environments^92,94,95^. Within these families, genera such as *Rhizobium*, *Klebsiella*, *Pseudomonas*, *Acinetobacter*, *Alcaligenes*, and *Bacillus* are recognized for their ability to increase plant health and growth while enabling plants to tolerate and remediate environmental pollutants^93,96^. *Bacillus* is one of the most prevalent bacterial endophytes^97^. This observation aligns with our findings, where members of the Bacillaceae family, including *Bacillus*, were frequently abundant across most experimental variants, except in those treated with paracetamol. Similarly, *Microbacterium*, a genus known for improving plant growth, inducing defense responses, and exhibiting strong antifungal properties^98^, was identified in the P + B variant. These results highlight the specificity of certain bacterial associations depending on the treatment conditions. Interestingly, some endophytic bacteria are frequently associated with fungicide treatments in plants. Yadav et al.^99^ reported an increased abundance of *Bacillus* in tomato plants treated with the fungicide propiconazole. Consistently, our study revealed that benomyl exposure led to notable enrichment of the endophytic bacterial community, including *Bacillus*, *Neobacillus*, and *Pantoea*. These genera are known to support plant health and growth through mechanisms such as nutrient acquisition, hormone production, and pathogen defense^80,100^. This enhanced bacterial community may result from the impact of fungicides on reducing competition for carbon and energy sources between bacteria and fungi, thereby promoting bacterial proliferation.

Overall, similar to the findings concerning the functional diversity of endophytes measured via the Biolog Ecoplate method, 16S rRNA analysis confirmed that the combined P + B treatment significantly enhanced the endophytic microbial community in zucchini. This treatment also promoted beneficial genera such as *Bacillus* and *Microbacterium*, which, according to the literature, support plant growth and remediation of a range of organic compounds^101–103^, with *Bacillus* having been shown to possess paracetamol-degrading capabilities^104^. In contrast, paracetamol alone reduced microbial diversity, highlighting its toxic effects and the specificity of bacterial responses to different treatment conditions.

## Conclusion

Our study demonstrates that zucchini efficiently absorbs paracetamol, accumulating in both roots and leaves, with leaf concentrations markedly higher when plants are exposed to a combination of paracetamol and benomyl. This suggests that benomyl may enhance the translocation of paracetamol within the plant, a phenomenon that warrants further investigation. Exposure to paracetamol caused significant reductions in biomass and alterations in chlorophyll content, indicating oxidative stress and impaired photosynthesis, consistent with previous studies on crop phytotoxicity at elevated paracetamol concentrations. Interestingly, co-application with benomyl mitigated some of these negative effects, improving biomass, chlorophyll levels, and phenolic compound accumulation, implying a potential protective role of benomyl. Paracetamol also significantly affected the functional diversity of endophytic microbial communities. Biolog EcoPlate™ assays showed reduced microbial metabolic activity, whereas combined treatment with benomyl enhanced microbial functional diversity, suggesting that benomyl may stimulate microbial activity even in the presence of pharmaceuticals.

Overall, these findings highlight the complex interactions between the studied substances, plant physiology, and microbial communities. While paracetamol poses risks to plant health and microbial diversity, combined applications with agrochemicals such as benomyl may improve plant resilience and microbial function in polluted soils. Further research is needed to clarify the mechanisms underlying these interactions and optimize their use in phytoremediation strategies.

## Supplementary Information

Below is the link to the electronic supplementary material.

Supplementary Material 1

## Data Availability

The dataset generated and analysed during the current study are available in University of Lodz repository DSpace http:/hdl.handle.net/11089/54535; the DNA sequences obtained during the current study are deposited in FigShare repository under following DOI: 10.6084/m9.figshare.30405640.
