# Supplementary material for: Benomyl modulates paracetamol bioaccumulation and endophytic microbiome diversity in zucchini
Source: Sci Rep. 2025 Dec 28;16:3807. doi: 10.1038/s41598-025-33977-6 (PMC12852693; doi:10.1038/s41598-025-33977-6)
Supplement: Supplementary file 1 — Supplementary Material 1 [file 41598_2025_33977_MOESM1_ESM.docx]

**Table S.1**: The time frame of benomyl spraying during the experiment; experimental variants: control (C), paracetamol (P), paracetamol with benomyl (P + B) and benomyl (B).

| Variant | 0 day | 5^th^ day | 12^th^ day | 17^th^ day | 19^th^ day | 24^th^ day | 26^th^ day |
| --- | --- | --- | --- | --- | --- | --- | --- |
| C | **-** | **-** | **-** | **-** | **-** | **-** | **-** |
| P | **-** | **-** | **-** | **-** | **-** | **-** | **-** |
| P + B | **-** | **-** | **+** | **+** | **-** | **+** | **-** |
| B | **-** | **-** | **+** | **+** | **-** | **+** | **-** |

“+” indicates spraying of benomyl; “-” indicates no benomyl treatment

**Table S.2**: Roots, stems and leaves biomass reduction percentages in plants variants: paracetamol (P), paracetamol with benomyl (P + B) and benomyl (B) compared to the control.

| Plants Variants | Biomass reduction percentages compared to control (%) | | |
| --- | --- | --- | --- |
|  | Roots | Stems | Leaves |
| P | 59 | 60 | 67 |
| P + B | 64 | 42 | 47 |
| B | 66 | 31 | 38 |

**Table S.3**: Chlorophyll a:b ratio for; *F* _(3, 8)_ = 48.48, *p* = 0.0001 for the variants. Lowercase letters indicate the significant difference at *p* ≤ 0.05 based on the Tukey post hoc test.

| Plants Variants | Chlorophyll a:b ratio (± SD, n = 3) |
| --- | --- |
| C | 1.17 ± 0.11 ^b^ |
| P | 2.10 ± 0.08 ^a^ |
| P +B | 1.94 ± 0.13 ^a^ |
| B | 1.98 ± 0.10 ^a^ |

**Table S.4**: CFU for leaf extraction (10^-1^ ) for variants; control (C), Paracetamol (P), paracetamol with benomyl (P + B) and benomyl (B).

| Variants | CFU* 10^2^/mL |
| --- | --- |
| C | 9.5 |
| P | ND |
| P + B | 25.5 |
| B | 12.5 |

ND: Not detected
